# Affordable CRISPR RNP‐Based Genome Editing in *Euglena gracilis*

**DOI:** 10.1002/cpz1.70357

**Published:** 2026-05-05

**Authors:** Anzu Minami, Minami Shimizu, Shun Tamaki, Vicki Nishinarizki, Yosua, Keiichi Mochida

**Affiliations:** ^1^ Center for Sustainable Resource Science RIKEN Yokohama Kanagawa Japan; ^2^ Kihara Institute for Biological Research Yokohama City University Yokohama Kanagawa Japan; ^3^ Department of Biological Science, Faculty of Sciences and Engineering Yasuda Women's University Asaminami‐ku Hiroshima Japan; ^4^ Department of Chemistry Faculty of Mathematics and Natural Sciences, Universitas Padjadjaran Jatinangor West Java Indonesia; ^5^ Research Center for Molecular Biotechnology and Bioinformatics Universitas Padjadjaran Bandung West Java Indonesia; ^6^ School of Information and Data Sciences Nagasaki University Nagasaki Japan

**Keywords:** Cas9 nucleases, CRISPR ribonucleoprotein (RNP)‐based genome editing, *Euglena gracilis*, transformation

## Abstract

Genome editing can enhance basic research and enable industrial applications of green algae. Here, we present an affordable, broadly applicable workflow for genome editing in the unicellular green alga *Euglena gracilis* using Cas9 nucleases. This method retains high editing efficiency while significantly lowering technical barriers. Unlike previous approaches that required specialized equipment, this protocol can be performed using a general‐purpose laboratory electroporator and a simplified clonal isolation procedure without the need for specialized micromanipulation devices. This protocol is compatible with a range of editing outcomes, such as targeted deletions and precise base substitutions, enabling more widespread genome editing in *Euglena*. © 2026 The Author(s). *Current Protocols* published by Wiley Periodicals LLC.

**Basic Protocol 1**: Culture of *Euglena gracilis*

**Basic Protocol 2**: sgRNA synthesis

**Basic protocol 3**: Transformation

**Basic protocol 4**: Genotyping

## INTRODUCTION

Genome editing has emerged as an indispensable tool in molecular genetics and strain engineering, enabling the direct interrogation of gene function and the rational modification of biological traits across diverse organisms (Chen et al., 2019; Jeong et al., 2023; Pacesa et al., 2024; Wang & Doudna, 2023). *Euglena gracilis*, a unicellular phytoflagellate capable of both photosynthetic and heterotrophic growth, has garnered substantial interest as a sustainable biological resource, with applications in food, cosmetics, and biofuel production (Harada et al., 2020). Until recently, however, this organism has largely remained genetically intractable, limiting both functional genetic analysis and targeted strain improvement. This barrier has now been surmounted through the delivery of a ribonucleoprotein (RNP) complex based on clustered regularly interspaced short palindromic repeats (CRISPR)‐associated nuclease (Cas), which enables efficient, transgene‐free genome editing in *E. gracilis* (Nomura et al., 2024). This technology has been used to reveal direct causal links between genotype and phenotype, as exemplified by studies of carotenoid biosynthesis and eyespot morphogenesis (Tamaki et al., 2023) and the unique noncanonical splicing codes of this alga (Nomura et al., 2025). RNP‐based genome editing has opened new avenues for the rational engineering of industrial strains with enhanced productivity and performance (Ishikawa et al., 2022; Nagamine et al., 2024).

Here, we present a genome‐editing workflow for *E. gracilis* that is highly efficient and can readily be employed in standard laboratory settings. Although our previous Cas9 RNP‐based approaches established the feasibility of transgene‐free mutagenesis in *E. gracilis*, they required specialized electroporation systems with finely tunable parameters and micromanipulation for single‐cell isolation (Nomura et al., 2020). This protocol removes these constraints, as it allows RNP delivery using conventional laboratory electroporators and clonal isolation without the need for micromanipulation. This workflow yields genome‐edited clones amenable to downstream analyses such as genotyping, assessment of editing efficiency, and phenotypic characterization at clonal resolution. We provide detailed protocols covering culture maintenance, single guide RNA (sgRNA) synthesis, RNP complex electroporation, cell plating, and genotype‐verified clonal isolation, establishing a practical framework for genome editing in *E. gracilis* for both fundamental research and industrial strain development.

*CAUTION*: When performing CRISPR RNP‐based genome editing, all microorganisms and reagents should be handled in accordance with institutional biosafety guidelines. Please be aware that many jurisdictions have specific regulations and restrictions regarding the creation and handling of genetically modified organisms (GMOs). Ensure compliance with all applicable laws and institutional requirements before beginning any work.

## STRATEGIC PLANNING

### Experimental schedule for *Euglena* genome editing

**Figure 1:**
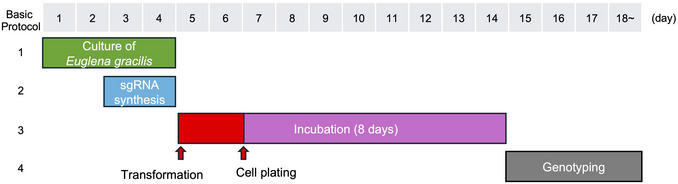
Experimental schedule for the *E. gracilis* genome‐editing protocol. Overview of the workflow and approximate timeline for Cas9 RNP‐mediated genome editing in *E. gracilis*. *E. gracilis* cells are cultured under standard growth conditions, followed by single guide RNA (sgRNA) synthesis and the delivery of the Cas9 ribonucleoprotein complex by electroporation. After electroporation, the cells are incubated for clonal outgrowth for approximately 10 days, after which putative genome‐edited clones are subjected to genotyping.

*NOTE*: All protocols involving animals must be reviewed and approved by the appropriate Animal Care and Use Committee and must follow regulations for the care and use of laboratory animals. Appropriate informed consent is necessary for obtaining and using human study material.

This protocol provides standard durations for each step (Fig. 1). Some cultivation steps are flexible and can be adjusted to accommodate the experimenter's schedule.

Days 1–4: Culture *E. gracilis* (4 days)
Days 3–4: sgRNA synthesis (2 days)
Day 5: Introduction of the RNP complex containing the sgRNA and Cas9 nuclease *via* electroporation
Days 7–14: Plating of electroporated *E. gracilis* cells on agar medium
and incubation in the light (to 8 days)
Day 15: Begin genotyping by genomic PCR

## Basic Protocol 1 CULTURE OF *EUGLENA GRACILIS*

This protocol describes the cultivation of *E. gracilis* strain Z under defined conditions to obtain healthy, actively growing cells suitable for genome editing experiments. Koren–Hutner (KH) medium is a commonly used defined medium for *E. gracilis* (Koren and Hutner, 1967). The medium is adjusted to a slightly acidic pH (pH 3.5) to suppress contamination by other microorganisms while maintaining optimal growth of *Euglena*. Cultures are maintained under continuous illumination at 26°C to promote photosynthetic growth, and cells in the logarithmic growth phase are used for electroporation‐based delivery of Cas9–sgRNA RNP complexes.

### Materials

*E. gracilis* strain Z (NIES‐48)
KH liquid medium, pH 3.5 (see recipe)

Sterile Erlenmeyer flasks, 100 ml (e.g., CTE33, Iwaki) fitted with silicone plugs
Sterile pipettes and tips, 200 µl
Sterile conical tube for measuring KH liquid medium, 50 ml
pH meter (e.g., pH Meter D‐52, HORIBA)
Laminar flow hood (LFH) (e.g., MCV‐161BNS, SANYO)
Autoclave for sterilization of media (e.g., LBS‐245, TOMY)
Incubator set to 26°C with 50 µmol photons m^−2^s^−1^ white light (e.g., Panasonic, MLR‐352H‐PJ)
Rotary shaker (e.g., multishaker MMS, EYELA) placed in the incubator

**Figure 2:**
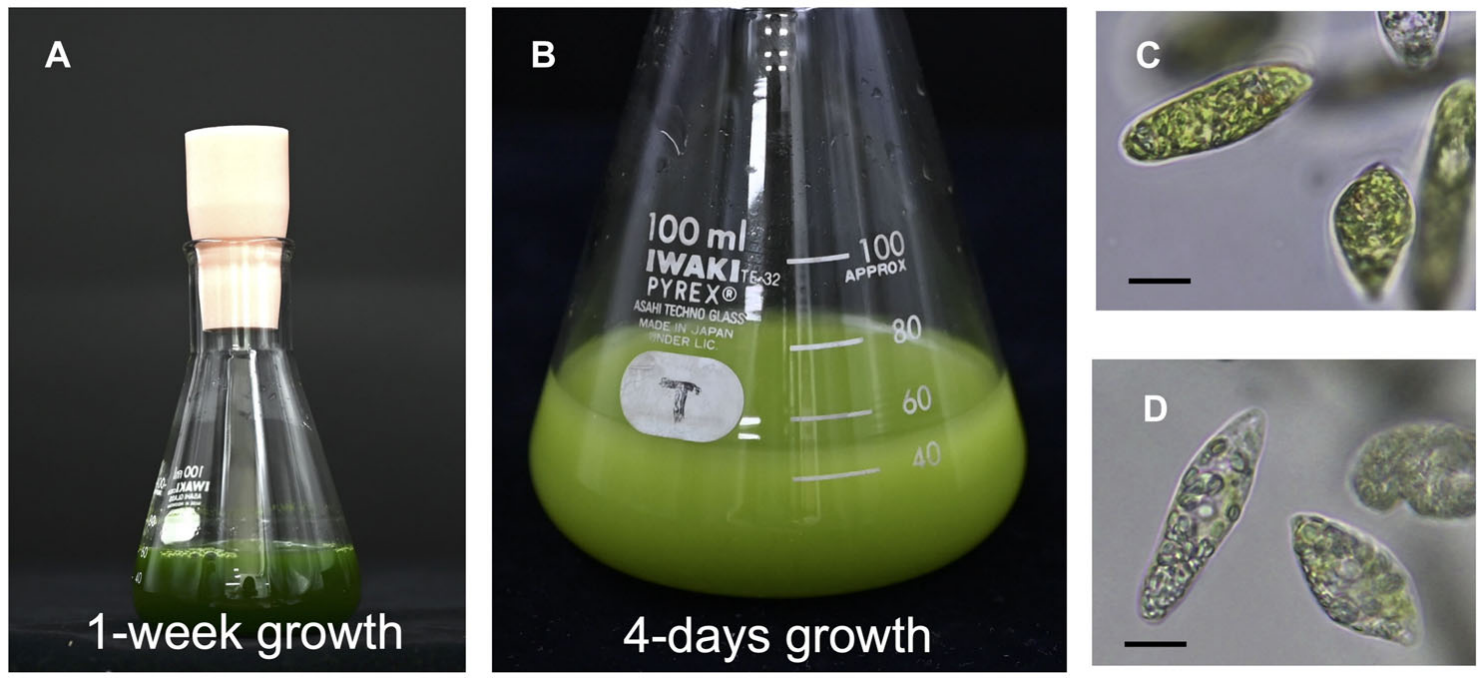
Liquid cultures of *Euglena gracilis* strain Z cells prepared for Cas9‐based RNP complex delivery. Representative photographs of *E. gracilis* strain Z cells cultured in KH liquid medium (pH 3.5) at 26°C under continuous light with rotary shaking. (**A, C**) 7‐day‐old cultured cells. Culture is maintained by routine weekly subculturing. (**B, D**) 4‐day‐old culture following inoculation, ready for electroporation for Cas9 RNP delivery. Scale Bars, 10 µm.

1 Culture *E. gracilis* strain Z in KH liquid medium, adjusted to pH 3.5 with potassium hydroxide, in an autoclaved sterile Erlenmeyer flask, on a rotary shaker (120 rpm) placed in the incubator at 26°C under continuous light (50 µmol photons m^−2^s^−1^) (Fig. 2).

2 Maintain the culture by inoculating 50 ml fresh KH liquid medium (measured in a sterile 50‐ml conical tube) with 100 µl from the old culture every week under a LFH.
3 Use *E. gracilis* cells cultured for 4 days for direct delivery of RNP complexes *via* electroporation.

## Basic Protocol 2 sgRNA SYNTHESIS

This protocol describes the synthesis of sgRNAs for CRISPR/Cas9 genome editing via a PCR‐based approach using a CUGA7 sgRNA synthesis kit. The method involves designing target‐specific sgRNAs, PCR amplification of sgRNA templates, and in vitro transcription to generate functional sgRNAs.

***Part A: sgRNA design, PCR amplification, and purification of a sgRNA template***

**Figure 3:**
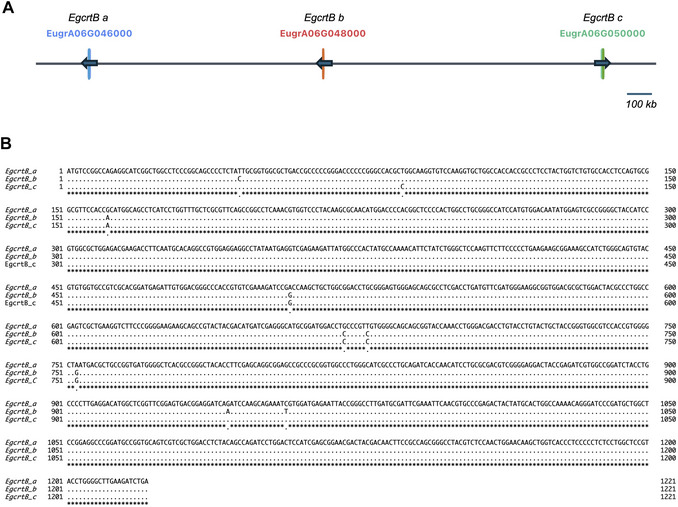
Genomic organization and sequence similarity of the three *EgcrtB* genes in the *E. gracilis* genome. (**A)** Genomic positions of the three *EgcrtB* loci (*EgcrtB a–c*) on chromosome 6. The *E. gracilis* genome contains three highly similar sequences putatively encoding crtB, located on the same chromosome and separated by large genomic intervals. These loci are not arranged in tandem; instead, they are dispersed along chromosome 6, with multiple unrelated genes located between each locus. (**B**) Multiple sequence alignment of the coding sequences of three *EgcrtB* sequences. Nucleotide sequence alignment reveals high sequence identity among the three *EgcrtB* genes.

The Cas9 nuclease is guided by sgRNAs and produces a double‐stranded break in the target site DNA. When using two sgRNAs targeting different positions within the same gene (sgRNA #1 and sgRNA #2), Cas9 can introduce double strand breaks at both sites. The endogenous DNA repair pathway of the cell ligates the released cut ends, deleting the intervening region. Based on this principle, two nonoverlapping sgRNA target regions are designed within the same gene, each containing a different target sequence, thereby generating deletion‐based knockout lines. The *E. gracilis* genome contains highly similar sequences on the same chromosome (Fig. 3). Because two or three homologous genes often contain extremely similar sequences, it is essential to verify all candidate 20‐nucleotide (nt) target sequences against the entire genome prior to final design. To generate complete knockout lines, sgRNAs must be designed to target all of the intended homologous genes.

### Materials

**Figure 4:**
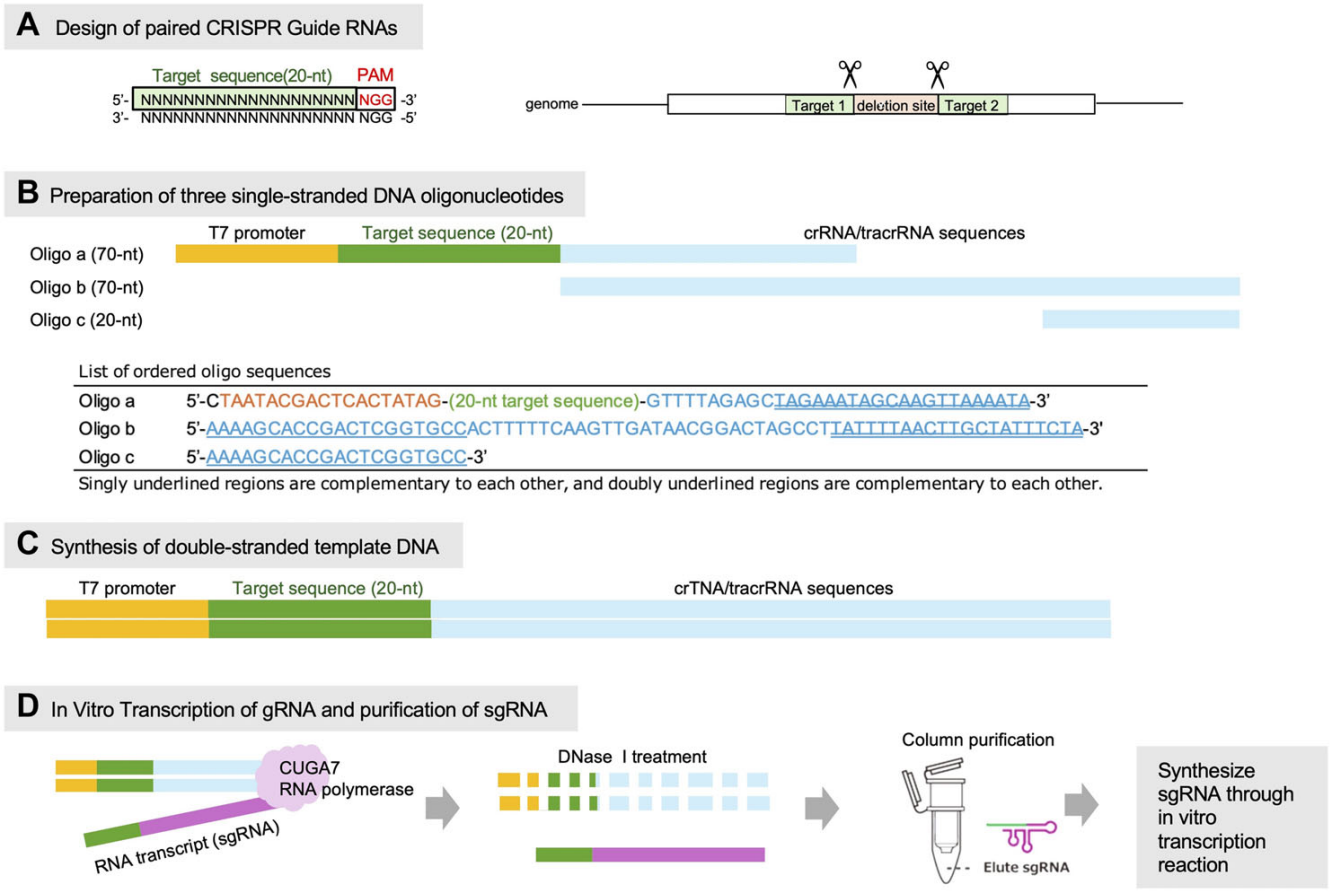
Overview of sgRNA design and sgRNA template assembly. (**A**) To excise the genomic fragment between the cut sites, two distinct single guide RNAs (sgRNAs) are designed to target the gene using typical CRISPR sgRNA design tools. (**B**, **C**) Three oligonucleotides for sgRNA synthesis are designed using a CUGA7 gRNA Synthesis Kit following the manufacturer's instructions. (**D**) In vitro transcription and purification of sgRNAs are carried out using a CUGA7 gRNA Synthesis Kit according to the manufacturer's instructions.

Three single‐stranded DNA oligonucleotides (refer to Fig. 4):
  Oligo a_1 [for sgRNA #1 sequence, oligonucleotide purification cartridge (OPC) purification]
  Oligo a_2 (for sgRNA #2 sequence, OPC purification)
  Oligo b [a part of the CRISPR RNA (crRNA)/trans‐activating crRNA (tracrRNA) sequence, OPC purification]
  Oligo c (a part of crRNA/tracrRNA sequence, OPC purification)
Agarose (*e.g*., Nippon Gene, cat. no. 312‐01193)
Tris‐acetate EDTA (TAE) buffer, 0.5× (see recipe)
DNA staining dye (*e.g*., FluoroVue DNA Fluorescent Staining Dye, SMOBIO)
Gel extraction kit (*e.g*., NucleoSpin Gel and PCR Clean‐up Kit, Takara Bio)
Milli‐Q water or nuclease‐free water
High‐fidelity PCR polymerase (*e.g*., Tks Gflex DNA Polymerase, Takara Bio)

PCR tubes, 0.2 ml
Sterile tubes, 1.5 ml
Thermal cycler (*e.g*., Veriti 96‐well thermal cycler 9902, Applied Biosystems)
Gel electrophoresis apparatus (*e.g*., Mupid One, Takara Bio)
Gel documentation system (*e.g*., LED‐Transilluminator, OPTIMA)
Spectrophotometer (*e.g*., NanoDrop One, Thermo Fisher Scientific)
Microcentrifuge
Pipettes and sterile tips, 10 and 200 µl

1 Collect information on the target gene from genomic databases and obtain the corresponding genomic sequence using datasets reported by Ebenezer et al. (2019) and Chen et al. (2024).
2 Search for candidate target regions in the genome. We recommend using commonly used tools such as Cas‐Designer (http://www.rgenome.net/cas‐designer/) to minimize off‐target effects. In the currently available *E. gracilis* genome assembly (Chen et al., 2024), highly homologous gene copies may be found within a single chromosomal assembly (Fig. 3). When designing sgRNAs simultaneously targeting homologs, the corresponding genomic regions are aligned to identify conserved sequences shared among them. For the generation of deletion mutants, two sgRNAs (sgRNA #1 and sgRNA #2) can be designed to target such conserved regions (Fig. 4A).
3 After identifying the two target sgRNA sequences (sgRNA #1 and sgRNA #2), design three oligonucleotides for each sgRNA using a CUGA7 gRNA synthesis kit according to the manufacturer's manual (https://www.nippongene.com/siyaku/product/genome‐editing/tds/tds‐hosoku‐r302_cuga7‐grna‐synthesis‐kit.pdf). For the target sequence incorporated into Oligo a, choose a 20‐nt region located immediately upstream of a 5′‐NGG protospacer‐adjacent motif (PAM), which is required by Cas9. Design the three oligonucleotides (Oligos a–c) for both sgRNA #1 and sgRNA #2, where Oligo b and Oligo c are common sequences, and Oligo a is target‐specific. Order all oligonucleotides with OPC purification or an equivalent RNA‐compatible grade (Fig. 4B).
4 Prepare two PCR reactions (50 µl each) for sgRNA #1 and sgRNA #2 in 0.2‐ml PCR tubes as follows:

**Table:** 

Gflex PCR buffer, 2 ×	25 µl
Oligo a (target‐specific), 10 µM	2 µl
Use Oligo a_1 for sgRNA #1 (Tube 1)	
Use Oligo a_2 for sgRNA #2 (Tube 2)	
Oligo b, 0.1 µM	2 µl
Oligo c, 10 µM	2 µl
Tks Gflex DNA polymerase (1.25 units)	1 µl
Water	18 µl
Total volume: 50 µl
  Oligo b and Oligo c are identical in both tubes; only Oligo a differs between the two reactions.
5 Run the PCR in a thermal cycler using the following program:

**Table:** 

Initial denaturation: 98°C	2 min
35 cycles: 98°C	10 s
55°C	15 s
68°C	30 s
Final extension: 68°C	5 min
Hold: 20°C
6 Load all PCR products onto a 2% (w/v) agarose gel prepared with 0.5 × TAE buffer and perform electrophoresis at 135 V for 30 min. Stain the gel with a DNA fluorescent staining dye for 15 min, visualize the bands using a gel documentation system, and excise the band corresponding to the expected PCR product (118 bp).
7 Purify the PCR product from the gel slice using the gel extraction kit following the manufacturer's instructions.
8 Elute the purified DNA in 20 µl of nuclease‐free water.
9 Measure the DNA concentration using a spectrophotometer. A total yield of ≥100 ng is sufficient (Fig. 4C).

***Part B: In vitro transcription and purification of sgRNAs***

This protocol describes the in vitro transcription and purification of sgRNAs using a CUGA7 gRNA Synthesis Kit. All reagents used in the reaction, including transcription buffer, DTT, NTP mix, nuclease‐free water, and enzyme solution, are provided in the kit. When performed correctly, this protocol yields 2000–4000 ng/µl of high‐quality sgRNA suitable for CRISPR/Cas9‐mediated genome editing.

***Materials***

Purified sgRNA template (from Part A, >100 ng)
sgRNA Synthesis Kit (*e.g*., CUGA7 sgRNA Synthesis Kit, Nippon Gene, Japan; cat. no. 314‐08691)
  CUGA7 Enzyme Solution, 50 µl × 1 bottle
  5×Transcription Buffer, 200 µl × 1 bottle
  0.1 M DTT, 100 µl × 1 bottle
  NTP mix, 300 µl × 1 bottle
  DNase I (RNase free), 100 µl × 1 bottle
  ddWater (RNase free), 1 ml × 5 bottles
  gRNA Binding Buffer, 30 ml × 1 bottle
  gRNA Wash Buffer, 40 ml × 1 bottle
  Spin Column, 50 pieces × 1 bag

PCR tubes, 0.2 ml
Thermal cycler (*e.g*., Veriti 96‐well thermal cycler 9902, Applied Biosystems)
Sterile centrifuge tubes, 1.5 ml
Microcentrifuge
Pipettes and sterile tips, 10 µl, 200 µl, and 1 ml
Spectrophotometer (*e.g*., NanoDrop One, Thermo Fisher Scientific)

#### Perform in vitro transcription (Fig. 4D)

1 Prepare two separate transcription reactions (20 µl each; one for sgRNA #1 and one for sgRNA #2) in 0.2‐ml PCR tubes by adding the following components:

**Table:** 

Transcription buffer, 5 ×	4 µl
DTT, 0.1 M	2 µl
NTP mix	6 µl
CUGA7 enzyme solution	1 µl
Purified gRNA template (≥ 100 ng)	7 µl^a^
Total volume: 20 µl
  Higher amounts of template DNA generally increase the yield of sgRNA.
2 Incubate the mixture at 37°C for 5–6 h in a thermal cycler.
3 Add 2 µl of DNase I to each reaction mixture and mix by pipetting.
4 Incubate at 37°C for 15 min in a thermal cycler to digest the template DNA.

#### Purify the sgRNA

5 Transfer each reaction mixture to a 1.5‐ml microcentrifuge tube and add 578 µl of sgRNA binding buffer. Mix by gentle inversion.
6 Transfer the mixture to the spin column provided in the kit.
7 Centrifuge at 13,000 × *g* for 1 min at 4°C and discard the flow‐through.
8 Add 750 µl of sgRNA wash buffer to the spin column.
9 Centrifuge at 13,000 × *g* for 1 min at 4°C and discard the flow‐through.
10 Centrifuge the column again at 13,000 × *g* for 2 min at 4°C to remove residual wash buffer.
11 Place the spin column into a new 1.5‐ml microcentrifuge tube.
12 Add 20 µl of ddWater directly on top of the membrane and incubate for 3 min at room temperature.
13 Centrifuge at 13,000 × *g* for 1 min at 4°C to elute the sgRNA.
14 Measure the sgRNA concentration using the spectrophotometer. Expected yield: 2000–4000 ng/µl.
15 Store the sgRNA samples at −80°C if they are not used immediately.

## Basic Protocol 3 TRANSFORMATION

This protocol describes the transformation of *E. gracilis* cells using Cas9‐containing RNP complexes, which are RNPs composed of Cas9 nuclease bound to the sgRNA and are delivered by electroporation. Log‐phase cells are harvested, washed, and combined with pre‐assembled RNPs containing Cas9, sgRNA #1, and sgRNA #2 to induce targeted deletions. Following electroporation under optimized conditions, the cells recover briefly in the liquid medium before being plated at low density on the solid medium. This approach enables efficient selection and growth of single colonies without cell sorting.

***Part A: Introduction of sgRNA and Cas9 nuclease by electroporation***

### Materials

Cramer‐Myers (CM) liquid medium, pH 5.5 (Cramer & Myers, 1952; see recipe)
Filter‐sterilized sucrose, 0.3 M (see recipe)
Two sgRNAs (from Basic Protocol 2 Part B, stored at −80°C)
Cas9 nuclease (Alt‐R S.p. Cas9 Nuclease V3, Integrated DNA Technologies; stored at −20°C)
*E. gracilis* cells in log‐phase growth (cultured in KH liquid medium, pH 3.5, for 4 days at 26°C)
KH liquid medium, pH 5.5 (see recipe)
NaCl, 5 M (see recipe)

LFH (*e.g*., MCV‐161BNS, SANYO)
  Procedures marked with (LFH) must be performed inside the LFH.
Sterile centrifuge tubes, 15 ml
PCR tubes, 0.2 ml
Sterile microcentrifuge tubes, 1.5 ml
Microscope (*e.g*., CX43, OLYMPUS)
Thoma hemocytometer (*e.g*., 177‐312C, Watson)
Microcentrifuge
Electroporation cuvette, 2‐mm gap (*e.g*., EC‐002S, NEPAGENE)
Gel electrophoresis apparatus (*e.g*., Mupid One, Takara Bio)
Gel documentation system (*e.g*., OPTIMA, LED‐Transilluminator)
Sterile culture tubes, 15 ml
Pipettes and sterile tips, 10 µl, 200 µl, and 1 ml
Rotary shaker

#### Prepare electroporation solution and RNP complexes

1 (LFH) Prepare 5 ml of electroporation solution in a sterile 15‐ml centrifuge tube.

**Table:** 

CM liquid medium, pH 5.5	3 ml
Sucrose, 0.3 M	2 ml
Final volume	5 ml
2 Prepare the two RNP complexes for sgRNA #1 and sgRNA #2 separately in 0.2‐ml PCR tubes (Fig. 5B).

**Table:** 

sgRNA	4000 ng
Cas9 nuclease	0.8 µl

**Figure 5:**
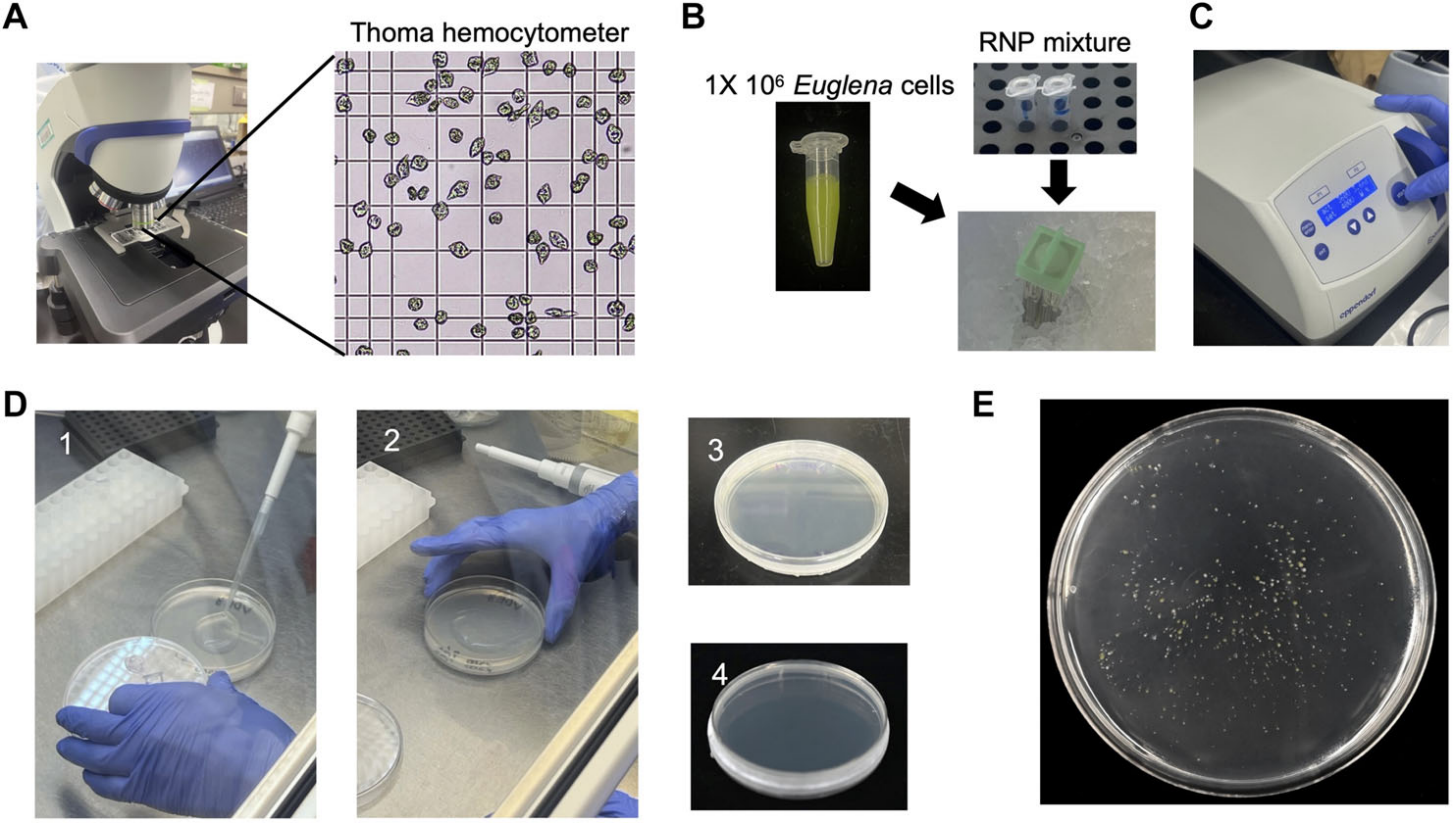
Electroporation and cell plating for Cas9 RNP‐mediated genome editing in *Euglena gracilis*. (**A**) Cell counting prior to electroporation. *E. gracilis* cells are observed under a light microscope and counted using a Thoma hemocytometer to determine cell titer. (**B**) Preparation of the mixture for electroporation. A total of 1 × 10^6^ *E. gracilis* cells are collected, resuspended in 1 ml of electroporation solution, and mixed with two Cas9–sgRNA RNP complexes. (**C**) Electroporation of *E. gracilis* cells. The cell–RNP complex mixture is transferred to an electroporation cuvette and subjected to electroporation at 400 V for 10 pulses using a general‐purpose electroporator. (**D**) Cell plating after electroporation. KH liquid medium is added to the cuvette immediately after electroporation. After incubation for 2 days in darkness, an arbitrary volume of cells is suspended in top agar. The cells are gently transferred to solid medium, evenly spread onto agar plates under sterile conditions, and incubated for 7–9 days. (**E**) Representative photograph of an agar plate after 7 days of incubation. After 7 days of incubation at 26°C in the light, individual colonies are selected for genotyping and clonal analysis.

3 Incubate both RNP complex mixtures at room temperature for 15 min to allow complex formation.

#### Prepare cells for electroporation

4 Use healthy *E. gracilis* cells in log‐phase growth cultured in KH liquid medium (pH 3.5) for 4 days at 26°C (from Basic Protocol 1).
5 (LFH) Transfer 20 µl of the cell suspension into a 1.5‐ml microcentrifuge tube.
6 (LFH) Add 1 µl of 5 M NaCl solution to the 20‐µl cell suspension and mix gently.
7 Count the cells under a microscope using the hemocytometer according to the manufacturer's instructions (Fig. 5A).
8 (LFH) Collect 1 × 10^6^ *E. gracilis* cells into sterile 1.5‐ml microcentrifuge tubes. The cells are typically divided into 4–5 tubes.

#### Wash cells

9 Centrifuge the *E. gracilis* cells at 5000 × *g* for 1 min at room temperature.
10 (LFH) Discard the supernatant. Add 1 ml of electroporation solution to the first tube to resuspend the pellet. Transfer this suspension sequentially to the remaining tubes to wash all cell pellets. Finally, combine all washed cells into a single tube.
11 Centrifuge at 5000 × *g* for 1 min at room temperature.
12 (LFH) Discard the supernatant and resuspend the cell pellet in 1 ml of electroporation solution.
13 Repeat steps 11–12 two more times (*i.e*., two washes).
14 (LFH) After the final wash, discard all of the supernatant and resuspend the cell pellet in 1 ml of electroporation solution (Fig. 5B).

#### Prepare electroporation mixtures

15 Place the electroporation cuvettes (2‐mm gap) on ice.
16 (LFH) Prepare the electroporation mixture (50 µl total) directly in the 0.2‐ml PCR tube containing one of the RNP complex mixtures:
  For the generation of deletion knockout strains using two RNPs:

**Table:** 

RNP mixture 1 (from Step 2 of Basic Protocol 3A)	*X* µl
RNP mixture 2 (from Step 2 of Basic Protocol 3A)	*Y* µl
*Euglena* cell suspension	50 − (*X* + *Y*) µl
Total volume:	50 µl
17 (LFH) Transfer the 50‐µl electroporation mixture to a pre‐chilled 2‐mm gap electroporation cuvette and keep it on ice.
18 Electroporate the cells using 400 V for 10 pulses (Fig. 5C).
19 (LFH) Add 1 ml of KH liquid medium (pH 5.5) to the cuvette immediately after electroporation to facilitate cell recovery.
20 (LFH) Transfer the cell suspension from the cuvette to a 15‐ml culture tube using a sterile pipette. Add 1 ml of KH liquid medium (final volume: 2 ml).
21 Culture the cell suspension on a rotary shaker (120 rpm) at 26°C for 2 days in darkness.

***Part B: Cell plating***

This section describes plating electroporated cells at low density to obtain isolated colonies for screening. Cells are mixed with top agar and plated onto solid medium, where they recover and form visible colonies after 7–9 days of incubation.

***Materials***

KH top agar, 0.2% (w/v) agar in KH liquid medium, pH 3.5 (see recipe)
Electroporated cells in 2 ml of KH liquid medium (from Basic Protocol 3 Part A)
KH liquid medium (pH 5.5; see recipe)
KH medium (pH 5.5; see recipe)

Microwave
LFH (*e.g*., MCV‐161BNS, SANYO)
  Procedures marked with (LFH) must be performed inside the LFH.
Sterile microcentrifuge tubes, 1.5 ml
Surgical tape (*e.g*., Micropore Surgical Tape 1530‐0, 3 M)
Incubator set at 26°C with 50 µmol photons m^−2^s^−1^ white light (*e.g*., MLR‐352H‐PJ, Panasonic)
PCR tubes, 0.2 ml
Sterile centrifuge tubes, 15 ml
Sterile culture tubes, 15 ml
Microcentrifuge
Pipettes and sterile tips, 10 µl and 1 ml

#### Prepare cells for plating

1 If the top agar has not yet melted, heat it in the microwave. Once it has melted, leave it at room temperature to cool down.
2 (LFH) Mix the electroporated cell suspension thoroughly to ensure even distribution.
3 (LFH) Add 200 µl of electroporated cell suspension and 600 µl of KH liquid medium (pH 5.5) into a 1.5‐ml tube.
4 (LFH) Prepare serial dilutions of cells in top agar. In separate sterile 1.5‐ml microcentrifuge tubes, prepare the following:
  Tube 1: 2 µl cell suspension + 1 ml top agar
  Tube 2: 4 µl cell suspension + 1 ml top agar
  Tube 3: 8 µl cell suspension + 1 ml top agar
5 Mix each tube thoroughly by gentle inversion to evenly distribute the cells.
  Prepare duplicate tubes for each dilution (total of 6 tubes) to ensure sufficient colonies for screening.
  Plate, incubate, and recover cells.
6 (LFH) Pour each cell–agar mixture onto a plate containing KH solid medium (pH 5.5) (Fig. 5D‐1).
7 (LFH) Gently swirl the plate to distribute the top agar evenly across the surface (Fig. 5D‐2).
8 (LFH) Seal the plate with surgical tape (Fig. 5D‐3).
9 Incubate the sealed plates at 26°C for 24–48 h under continuous white light with a light flux of 50 µmol photons m^−2^s^−1^.
10 After the agar has fully solidified, invert the plate so that the solid medium is facing upward (Fig. 5D‐4).
11 Incubate at 26°C for 7–9 days under continuous white light with a light flux of 50 µmol photons m^−2^s^−1^ until visible colonies appear (Fig. 5E).
  Colonies typically become visible after 6 days. Check plates daily after day 7 for colony formation.

## Basic Protocol 4 GENOTYPING

Because *E. gracilis* may possess highly similar gene copies within its genome, genomic PCR primers should be designed to account for sequence similarity among homologs. In addition, sequence variation may be found between published reference assemblies and wild‐type (WT) strains used for genome editing. Therefore, genomic sequences encompassing the target sites should be verified in the WT strains used prior to analyzing genome‐edited sequences. For individual lines in which gene editing has been confirmed by genomic PCR, the precise edited site should ideally be verified by cloning and Sanger sequencing.

**Figure 6:**
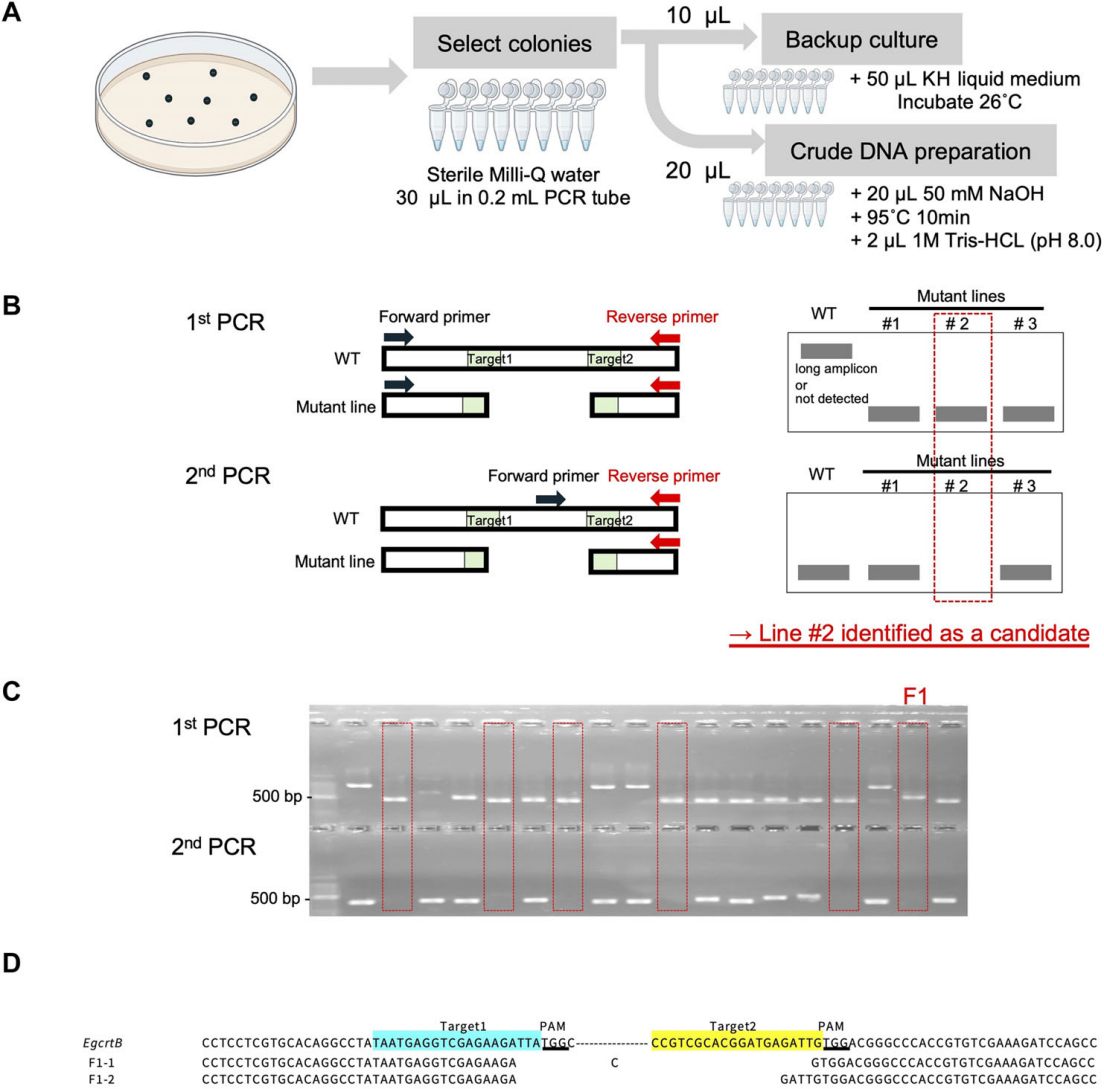
Two‐step PCR‐based genotyping strategy for detecting sgRNA‐induced deletions. (**A**) DNA extraction method using a picked colony and preparation of a backup culture. (**B**) Diagram of the two‐step PCR‐based approach to identify genomic deletions generated by pairs of sgRNAs. In the first PCR, primers are positioned outside the two sgRNA target sites to amplify across the expected deletion region; regions with a deletion are detected as shorter amplicons compared to the wild‐type (WT) amplicon. In the second PCR, primers are located within the region deleted by sgRNA‐targeted cleavage; deletion events are confirmed based on the absence of an amplicon. Line #2 shows the expected deletion band in the first PCR and no WT amplification in the second PCR and was therefore selected for cloning. (**C**) Genotyping result of *EgcrtB* using a two‐step PCR‐based approach. Red boxes indicate candidate lines exhibiting the expected deletion pattern described in (**B**). Among these candidates, Line #F1 was selected for further analysis. The primers used are listed in the Supplementary Table 1. (**D**) Nucleotide sequence alignment of PCR fragments amplified from the WT and Line #F1. The genomic regions encompassing the gRNA target sites in Line #F1 were amplified by genomic PCR, cloned, and colony PCR products were subjected to Sanger sequencing. Multiple sequence variations were identified among the amplicons from Line #F1, including nucleotide deletions. Two gRNA target sequences are highlighted in yellow and blue, respectively. The protospacer adjacent motif (PAM) is shown adjacent to each gRNA target sequence.

First, a primer set flanking the deletion region generated by the two sgRNAs should be designed and used for genotyping PCR (1^st^ PCR) (Fig. 6B). Next, to determine whether the deletion is present in all homologous genes, a second PCR (2^nd^ PCR) should be performed using primers designed within the deleted region. For colonies in which the deletion is confirmed by the first PCR and the WT region is not amplified in the second PCR, proceed with cloning and sequencing. The primers should be redesigned if nonspecific products are frequently amplified or if clear PCR products cannot be obtained. Alternatively, gene‐specific primers can be designed to sequence each homolog individually.

Obtaining sufficient colonies for DNA preparation from genome‐edited cells plated on agar medium typically requires approximately 10 days. To rapidly evaluate genome editing by the designed sgRNAs, leftover electroporated cells that were not plated on agar can be used. These cells will be cultured for several days at 26°C under continuous white light (50 µmol photons m^−2^s^−1^) with shaking. Genomic DNA can then be prepared using the simplified method described in Basic Protocol 4, and genotyping by the first PCR (1^st^ PCR) can be performed to assess the presence of deletion‐type edits.

***Part A: Simple DNA extraction by the alkaline lysis method***

This rapid alkaline lysis method extracts crude genomic DNA suitable for PCR‐based genotyping while preserving a backup culture of each colony.

### Materials

*E. gracilis* colonies on KH solid plates (from Basic Protocol 3 Part B)
Sterile Milli‐Q water or nuclease‐free water
KH liquid medium, pH 3.5 (see recipe)
NaOH, 50 mM (avoid prolonged storage; see recipe)
*E. gracilis* WT strain (control for mutant screening)
Tris·HCl, 1 M (pH 8.0; see recipe)

8‐lane PCR tubes, 0.2 ml
LFH (*e.g*., MCV‐161BNS, SANYO)
Autoclaved toothpicks
Refrigerator
Pipettes and sterile tips, 10 µl, 200 µl
PCR tubes, 0.2 ml
Incubator set at 26°C with 50 µmol photons m^−2^s^−1^ white light (*e.g*., MLR‐352H‐PJ, Panasonic)
Vortex mixer (*e.g*., Vortex‐Genie2, Scientific Industries)
Thermal cycler (*e.g*., Veriti 96‐well Thermal cycler 9902, Applied Biosystems)
Sterile microcentrifuge tubes, 1.5 ml
Microcentrifuge

#### Select and prepare colonies

1 Confirm that *Euglena* colonies are growing on the solid plates. Select plates with well‐isolated colonies suitable for picking.
2 (LFH) Add 30 µl of sterile Milli‐Q water to each well of an 8‐lane 0.2‐ml PCR tube (Fig. 6A).
  Process colonies in batches of 24 (3 × 8‐lane tubes).
3 (LFH) Pick a *Euglena* colony with a sterile toothpick and resuspend it in water in a PCR tube well.
4 Preserve the original colony plates upside down at 4°C (refrigerator) as master stock for recovering positive clones.

#### Create a backup culture

5 (LFH) Resuspend the cells by pipetting and transfer 10 µl of the suspension to a new PCR tube (“culture tube”).
6 (LFH) Add 50 µl of KH liquid medium (pH 3.5) to the culture tube and cap securely.
7 Place the culture tube upright in a tube rack and incubate at 26°C under continuous light (50 µmol photons m^−2^s^−1^) in an incubator. If subculturing is required, please do so within 1 month.
*These backup cultures can be expanded if successful editing is confirmed by genotyping*.

#### Perform alkaline lysis

8 To the remaining 20 µl of the cell suspension in the original tube (“lysis tube”), add 20 µl of 50 mM NaOH and mix thoroughly by vortexing.
9 For the WT strain, transfer 10 µl of *Euglena* WT culture to a 0.2‐ml PCR tube and add 190 µl of Milli‐Q water. Centrifuge at 16,000 × *g* for 2 min and completely remove the supernatant. Resuspend the pellet in 20 µl of Milli‐Q water. Then add 20 µl of 50 mM NaOH and mix thoroughly by vortexing, as described above.
10 Incubate the lysis tube at 95°C for 10 min and cool down to room temperature using a thermal cycler.
11 Add 2 µl of 1 M Tris·HCl (pH 8.0) to neutralize the mixture and vortex briefly.
12 Centrifuge at 16,000 × *g* for 5 min at 4°C.
13 Transfer 30 µl of the supernatant to a new 8‐lane 0.2‐ml PCR tube. Use 0.5–2 µl of the supernatant (“crude DNA preparation”) as the template for PCR genotyping (Basic Protocol 4 Part B). Store the remaining lysate at −20°C for future genotyping applications.

***Part B: PCR amplification for genotyping (Fig. 6B)***

This protocol describes the two‐step PCR strategy for screening edited colonies. Prior to large‐scale screening, validate both primer sets using 8–16 test samples to confirm specific amplification.

***Materials***

OmniPCR Supermix (OnePCR Ultra) with Fluorescent Dye (GeneDireX)
Primer set for the 1^st^ PCR (flanking the deletion region)
Primer set for the 2^nd^ PCR (within the deletion region; amplifies only in WT)
Crude genomic DNA (from Basic Protocol 4 Part A)
DNA marker premixed with DNA staining dye (*e.g*., 500 bp DNA Ladder, Takara Bio; FluoroDye DNA Fluorescent Loading Dye Green, SMOBIO)
Milli‐Q water or nuclease‐free water
1%–1.5% agarose gel in TAE buffer

Sterile tubes, 1.5 ml
PCR tubes, 0.2 ml
Thermal cycler (*e.g*., Veriti 96‐well Thermal cycler 9902, Applied Biosystems)
Pipettes and sterile tips, 10 µl, 200 µl, and 1 ml
Gel electrophoresis apparatus (*e.g*., Mupid One, Takara Bio)
Gel documentation system (*e.g*., LED‐Transilluminator, OPTIMA)

#### Perform 1^st^ PCR (deletion screening)

1 Prepare a PCR mixture (final volume 10 µl) as follows:

**Table:** 

OmniPCR	5 µl
10 µM Primer F	0.2 µl
10 µM Primer R	0.2 µl
Crude DNA preparation	0.5–1.0 µl
Milli‐Q water	10 µl
2 Run the PCR in a thermal cycler using the following program:

**Table:** 

Initial denaturation:	98°C	5 min
35–40 cycles:	94–98°C	20–30 s
	56–68°C	1 min
	72°C	2 min
Final extension:	72°C	5 min
Hold:	15°C
  Optimize the annealing temperature (56–68°C) based on the Tm of the primers.
3 Analyze 5 µl of the PCR product by electrophoresis on a 1%–1.5% (w/v) agarose gel in 0.5 × TAE buffer.
4 Image the gel using a gel documentation system
5 Identify colonies showing a deletion band.

#### Perform 2^nd^ PCR (WT/non‐deletion screening)

6 For colonies that were positive in the 1^st^ PCR, repeat steps 1–5 using the 2^nd^ primer set (non‐deletion region).
7 Select candidate knockout colonies based on the following criteria:
  Presence of a deletion band in the 1^st^ PCR
  Absence of amplification in the 2^nd^ PCR
8 Proceed with cloning and sequencing (Basic Protocol 4 Part C) for selected colonies.

***Part C: Cloning and sequence verification***

Because sequence variation may exist between published reference assemblies and the WT strain used, the genomic sequence of the WT strain should be used as the reference when genotyping edited clones. The *E. gracilis* genome typically contains 2–3 copies of each gene, and these copies have high sequence similarity. In this genotyping experiment, as sequences that commonly recognize these homologous genes were used, the resulting PCR products must be cloned into a vector to sequence individual clones and distinguish them. If the PCR fragment is too short and does not contain regions that can differentiate among the homologous sequences, primers should be redesigned to amplify a longer region that includes the deletion. PCR should then be performed again to obtain a fragment suitable for cloning. Direct sequencing of PCR products is often difficult due to the presence of mixed amplicons from the different homologous genes. Therefore, it is preferable to use a kit that allows direct cloning of PCR products (*e.g*., pJET, CloneJET PCR Cloning Kit, and Thermo Fisher Scientific). Please follow the instructions provided in the original protocol, with the option to scale down the reaction if necessary.

After cloning, the PCR products obtained by colony PCR can be purified and used directly for Sanger sequencing. If a PCR polymerase containing fluorescent dyes (such as OmniPCR SuperMix) is used for colony PCR, the dyes can interfere with Sanger sequencing. Therefore, we recommend using a purified PCR product for sequencing. Since only a small amount of PCR product is needed, a scaled‐down purification step can be performed using magnetic beads. When PCR samples are amplified using a polymerase without fluorescent dyes, enzyme‐based cleanup methods such as ExoSAP treatment can be used prior to Sanger sequencing. Sequencing 15–20 clones per colony is essential to determine whether the deletion has occurred in all homologous genes. If only clones derived from the same gene are obtained, the genotyping primers may need to be redesigned.

## REAGENTS AND SOLUTIONS

*Use deionized distilled water in all the recipes and protocol steps*.

### CM liquid medium

Dissolve the following CM base components in distilled or deionized water:CM trace metal stock A (× 1000; store at 4°C) and CM vitamin stock B (× 1000; store at 4°C). Adjust the pH to 3.5 using 10% (v/v) sulfuric acid (H_2_SO_4_) and fill to a final volume of 1 L. Sterilize by autoclaving at 121°C for 20 min. Store at room temperature in the dark for up to 3 months.

### CM base components

Diammonium hydrogen phosphate [(NH_4_)_2_HPO_4_], 1 g/L
Monopotassium phosphate **(**KH_2_PO_4_), 1 g/L
Magnesium sulfate heptahydrate (MgSO_4_·7H_2_O), 0.2 g/L
Calcium chloride (CaCl_2_), 0.02 g/L

### CM trace metal stock A (× 1000, store at 4°C)

Prepare a total volume of 200 ml. Add 1 ml/L of medium.

Iron(II) sulfate heptahydrate (FeSO_4_·nH_2_O), 0.6 g/200ml (final concentration 3 mg/L)
Manganese(II) chloride tetrahydrate (MnCl_2_·4H_2_O), 0.36 g/200 ml (final concentration 1.8 mg/L)
Zinc sulfate heptahydrate (ZnSO_4_·7H_2_O), 0.08 g/200 ml (final concentration 0.4 mg/L)
Cobalt(II) sulfate heptahydrate (CoSO_4_·7H_2_O), 0.3 g/200 ml (final concentration 1.5 mg/L)
Sodium molybdate dihydrate (Na_2_MoO_4_·2H_2_O), 0.04 g/200 ml (final concentration 0.2 mg/L)
Copper(II) sulfate pentahydrate (CuSO_4_·5H_2_O), 0.004 g/200 ml (final concentration 0.02 mg/L)

### CM vitamin stock B (× 1000, store at 4°C)

Prepare in a 200 ml total volume. Add 1 ml/L of medium.

Thiamine hydrochloride (Vitamin B_1_; C_12_H_17_ClN_4_OS·HCl), 0.02 g/200 ml (final concentration 0.1 mg/L)
Cyanocobalamin (Vitamin B_12_; C_63_H_88_CoN_14_O_14_P), 0.1 mg/200 ml (final concentration 0.0005 mg/L)

### EDTA, 0.5 M, pH 8.0

Dissolve 93.06 g EDTA·2Na (Wako, cat. no. 343‐01861, MW 372.24) in approximately 400 ml of Milli‐Q water with stirring. Adjust the pH to 8.0 using NaOH solution (Nacalai Tesque, cat. no. 31511‐05). Bring the final volume to 500 ml with Milli‐Q water. Autoclave at 121°C for 20 min.

### KH liquid medium, pH 3.5 or 5.5

Dissolve the following KH medium base components in distilled or deionized water. Add KH trace metal stock A and KH trace metal stock B (× 1000; store at 4°C), and KH vitamin stock C (× 1000; store at 4°C). Adjust the pH to 3.5 or 5.5 using KOH and fill to a final volume of 1 L. Sterilize by autoclaving at 121°C for 20 min. Store at room temperature in the dark for up to 3 months.

### KH medium base components

l(+)‐Arginine hydrochloride (C_6_H_14_N_4_O_2_·HCl), 0.5 g/L
l‐Aspartic acid (C_4_H_7_NO_4_), 0.3 g/L
d(+)‐Glucose (C₆H_12_O_6_),12 g/L
l‐Glutamic acid (C_5_H_9_NO_4_), 4 g/L
Glycine [amino acetic acid; NH_2_CH_2_COOH (C_2_H_5_NO_2_)], 0.3 g/L
l‐Histidine hydrochloride monohydrate (C_6_H_9_N_3_O_2_·HCl·H_2_O), 0.05 g/L
dl‐Malic acid (C_4_H_6_O_5_), 6.5 g/L
Trisodium citrate dihydrate (Na_3_C_6_H_5_O_7_·2H_2_O), 0.5 g/L
Disodium succinate (Na_2_C_4_H_4_O_4_), 0.1 g/L
Ammonium sulfate [(NH_4_)_2_SO_4_], 0.5 g/L
Ammonium hydrogen carbonate (NH_4_HCO_3_), 0.25 g/L
Potassium dihydrogen phosphate (KH_2_PO_4_), 0.25 g/L
Calcium carbonate (CaCO_3_), 0.12 g/L
Magnesium carbonate hydroxide [(MgCO_3_)_4_Mg(OH)_2_·xH_2_O], 0.6 g/L

### KH trace metal stock A (× 1000, store at 4°C)

Prepare a total volume of 200 ml. Add 1 ml/L of medium.

EDTA‐2Na (disodium EDTA dihydrate; C_10_H_14_N_2_Na_2_O_8_·2H_2_O), 10 g/200 ml (final concentration 50 mg/L)
Ammonium iron (II) sulfate hexahydrate [(NH_4_)_2_Fe(SO_4_)_2_·6H_2_O], 10 g/200 ml (final concentration 50 mg/L)

### KH trace metal stock B (× 1000, store at 4°C)

Prepare a total volume of 200 ml. Add 1 ml/L of medium.

Manganese(II) sulfate pentahydrate (MnSO_4_·5H_2_O), 3.6 g/200 ml (final concentration 18 mg/L)
Zinc sulfate heptahydrate (ZnSO_4_·7H_2_O), 5 g/200 ml (final concentration 25 mg/L)
Ammonium molybdate tetrahydrate [(NH_4_)_6_Mo_7_O_24_·4H_2_O], 0.8 g/200 ml (final concentration 4 mg/L)
Copper(II) sulfate pentahydrate (CuSO_4_·5H_2_O), 0.24 g/200 ml (final concentration 1.2 mg/L)
Boric acid (H_3_BO_3_), 0.12 g/200 ml (final concentration 0.6 mg/L)
Cobalt(II) sulfate heptahydrate (CoSO_4_·7H_2_O), 0.1 g/200 ml (final concentration 0.5 mg/L)
Nickel(II) sulfate hexahydrate (NiSO_4_·6H_2_O), 0.1 g/200 ml (final concentration 0.5 mg/L)
Ammonium vanadate (V) (NH_4_VO_3_), 0.1 g/200 ml (final concentration 0.5 mg/L)

### KH vitamin stock C (× 1000, store at 4°C)

Prepare a total volume of 200 ml. Add 1 ml/L of medium.
Thiamine hydrochloride (Vitamin B_1_; C_12_H_17_ClN_4_OS·HCl), 0.5 g/200 ml (final concentration 2.5 mg/L)
Cyanocobalamin (Vitamin B_12_; C_63_H_88_CoN_14_O_14_P), 1 mg/200 ml (final concentration 0.005 mg/L)

### KH solid medium, pH 5.5 (0.8% agar)

To 500 ml of KH medium (pH 5.5, see recipe), add 4 g agar powder (0.8% w/v, Wako, cat. no. 016‐11875). Autoclave at 121°C for 20 min (TOMY, LBS‐245). Cool to approximately 55°C and pour into sterile petri dishes. Allow to solidify at room temperature, and store plates inverted at 4°C for up to 3 months.

### KH top agar medium, pH 3.5 (0.2% agar)

To 500 ml of KH medium (pH 3.5, see recipe), add 1 g agar powder for plants (0.2% w/v, Wako, cat. no. 016‐11875). Autoclave at 121°C for 20 min. Store at room temperature in the dark for up to 3 months. Before use, melt the agar by heating and maintain it at approximately 42–45°C for plating.

### NaCl, 5 M

Dissolve 14.6 g sodium chloride (Nacalai Tesuque, cat. no. 31320‐05) in Milli‐Q water and adjust to a final volume of 50 ml. Autoclave at 121°C for 20 min. Store at room temperature.

### NaOH, 50 mM

Dissolve 0.2 g sodium hydroxide (Nacalai Tesque, cat. no. 31511‐05) in Milli‐Q water and adjust to a final volume of 100 ml. Sterilize by filtration through a 0.2‐µm filter (ADVANTEC, cat. no. 39125221). Store at room temperature. Avoid long‐term storage.

### Sucrose solution, 0.3 M (sterile)

Dissolve 51.3 g sucrose (Nacalai Tesque, cat. no. 30404‐45) in Milli‐Q water and adjust to a final volume of 500 ml. Sterilize by filtration through a 0.2‐µm filter (ADVANTEC, cat. no. 39122120). Store at 4°C.

### TAE buffer, 0.5 × (working solution)

Dilute 50 ml of 50 × TAE buffer with 4950 ml Milli‐Q water. Store at room temperature.

### TAE buffer, 50 ×

Dissolve 121 g Tris base (Nacalai Tesque, cat. no. 35434‐21) in approximately 300 ml of Milli‐Q water. Add 28.5 ml glacial acetic acid (Wako, cat. no. 017‐00256) and 50 ml of 0.5 M EDTA, pH 8.0. Adjust to a final volume of 500 ml with Milli‐Q water. Store at room temperature.

### Tris·HCl, 1 M, pH 8.0

Dissolve 60.55 g Tris base (Nacalai Tesque, cat. no. 35434‐21) in approximately 400 ml of Milli‐Q water. Adjust pH to 8.0 with concentrated HCl. Adjust to a final volume of 500 ml with Milli‐Q water. Autoclave at 121°C for 20 min. Store at room temperature.

## Commentary

### Critical Parameters

The *E. gracilis* genome may contain two to three highly homologous copies per gene, which can make genotyping more time‐consuming than is typical in other organisms. Even when one of these copies carries a deletion, PCR amplification of the WT sequences derived from the remaining copies can still occur. Consequently, sequencing is necessary to confirm that all copies have been knocked out, which requires careful comparison of genomic sequences to identify copy‐specific differences and the design of sequencing primers targeting these differences. When technical issues such as poor amplification arise during genotyping, the primers may preferentially amplify nonspecific fragments; in such cases, redesigning the primers is often more efficient and may help minimize overall time loss. Table 1 presents the typical issues encountered and the possible solutions.

**Table 1:** Troubleshooting Guide for Transformation of *Euglena gracilis*

Problem	Possible cause	Solution
Few colonies on the plate	Cells not in good condition	Handle the *Euglena* cells gently with a pipette. It is also a good idea to check under a microscope that the cells are not crushed. Further, after electroporation, adjust the number of cells spread on the plate.
PCR‐based genotyping does not work	Primer design issues	In many cases, the problem can be resolved by changing the primers. In particular, designing primers near repetitive sequences often results in poor amplification and should be avoided. Avoiding repetitive regions, paying attention to GC content, and using dedicated primer design tools are highly effective strategies for improving reaction efficiency and specificity. Additionally, if amplification yield is low or multiple nonspecific bands are observed, increasing the annealing temperature may improve the results. In some cases, adding 1 M betaine to the PCR reaction buffer may improve amplification efficiency.

### Understanding Results

In this protocol, successful genome editing of *EgcrtB* is evaluated using a two‐step PCR‐based genotyping strategy (Fig. 6C) followed by nucleotide sequence analysis of PCR products (Fig. 6D). Three *EgcrtB* loci located on chromosome 6 of *E. gracilis* (Fig. 3) were targeted. Two different gRNAs were designed to recognize these loci, and transformation was performed using the corresponding sgRNAs. Primers used for genotyping are listed in Supplementary Table S1.

In the first step of genotyping, primers positioned outside the two sgRNA target sites were used to amplify across the expected deletion region. When deletions were introduced, PCR products shorter than the WT amplicon were detected. In the second step, a primer within a region expected to be deleted was paired with a primer from the first PCR; loss of amplification indicated successful deletion (Fig. 6C). Correctly edited strains therefore exhibit a deletion band in the first PCR and no amplification in the second PCR.

For the *EgcrtB* knockout mutant #F1, the gRNA target region was amplified by genomic PCR and cloned into a plasmid vector. Colony PCR products from the clone colonies were then subjected to Sanger sequencing, which confirmed deletions at the gRNA target sites and adjacent protospacer adjacent motif (PAM) sequences (Fig. 6D). Multiple nucleotide deletions spanning the gRNA target regions were identified in the sequenced colony PCR products, likely representing independent editing events in different homologous copies.

### Time Considerations

Please refer to Figure 1 for details.

### Author Contributions

**Anzu Minami**: Conceptualization; investigation; writing—original draft; writing—review and editing. **Minami Shimizu**: Conceptualization; investigation. **Shun Tamaki**: Conceptualization; investigation; writing—original draft. **Vicki Nishinarizki**: Investigation. **Yosua**: Investigation. **Keiichi Mochida**: Conceptualization; writing—original draft; writing—review and editing; funding acquisition.

### Conflict of Interest

The authors declare no conflict of interest. All authors approved the final manuscript.

## Supporting information

Supplementary Table S1: Sequences of oligonucleotides used for genome editing of EgcrtB

## Data Availability

All data generated or analyzed during this study are included in this article.
